# Myocardial Structural Alteration and Systolic Dysfunction in Preclinical Hypertrophic Cardiomyopathy Mutation Carriers

**DOI:** 10.1371/journal.pone.0036115

**Published:** 2012-05-04

**Authors:** Kai Hang Yiu, Douwe E. Atsma, Victoria Delgado, Arnold C. T. Ng, Tomasz G. Witkowski, See Hooi Ewe, Dominique Auger, Eduard R. Holman, Anneke M. van Mil, Martijn H. Breuning, Hung Fat Tse, Jeroen J. Bax, Martin J. Schalij, Nina Ajmone Marsan

**Affiliations:** 1 Department of Cardiology, Leiden University Medical Center, Leiden, The Netherlands; 2 Cardiology Division, Department of Medicine, Queen Mary Hospital, University of Hong Kong, Hong Kong; 3 The Interuniversity Cardiology Institute of the Netherlands, Utrecht, The Netherlands; 4 Department of Human and Clinical Genetics, Leiden University Medical Center, Leiden, The Netherlands; Sapienza University of Rome, Italy

## Abstract

**Background:**

To evaluate the presence of myocardial structural alterations and subtle myocardial dysfunction during familial screening in asymptomatic mutation carriers without hypertrophic cardiomyopathy (HCM) phenotype.

**Methods and Findings:**

Sixteen HCM families with pathogenic mutation were studied and 46 patients with phenotype expression (Mut+/Phen+) and 47 patients without phenotype expression (Mut+/Phen−) were observed. Twenty-five control subjects, matched with the Mut+/Phen− group, were recruited for comparison. Echocardiography was performed to evaluate conventional parameters, myocardial structural alteration by calibrated integrated backscatter (cIBS) and global and segmental longitudinal strain by speckle tracking analysis. All 3 groups had similar left ventricular dimensions and ejection fraction. Basal anteroseptal cIBS was the highest in Mut+/Phen+ patients (−14.0±4.6 dB, p<0.01) and was higher in Mut+/Phen− patients as compared to controls (−17.0±2.3 vs. −22.6±2.9 dB, p<0.01) suggesting significant myocardial structural alterations. Global and basal anteroseptal longitudinal strains (−8.4±4.0%, p<0.01) were the most impaired in Mut+/Phen+ patients as compared to the other 2 groups. Although global longitudinal strain was similar between Mut+/Phen− group and controls, basal anteroseptal strain was lower in Mut+/Phen− patients (−14.1±3.8%, p<0.01) as compared to controls (−19.9±2.9%, p<0.01), suggesting a subclinical segmental systolic dysfunction. A combination of >−19.0 dB basal anteroseptal cIBS or >−18.0% basal anteroseptal longitudinal strain had a sensitivity of 98% and a specificity of 72% in differentiating Mut+/Phen− group from controls.

**Conclusion:**

The use of cIBS and segmental longitudinal strain can differentiate HCM Mut+/Phen− patients from controls with important clinical implications for the family screening and follow-up of these patients.

## Introduction

Hypertrophic cardiomyopathy (HCM) is the most common inherited cardiac disease and is the leading cause of sudden cardiac death in young individuals. [1] It is caused by genetic mutations encoding sarcomere proteins and the clinical diagnosis is characterized by unexplained left ventricular hypertrophy (LVH). However, HCM phenotypic expression is extremely variable and some patients may show only mild LVH or normal left ventricular (LV) thickness. [2], [3] Genetic testing for pathogenic mutations allows for a certain diagnosis and identification of HCM mutation carriers before, and independent of, the development of LVH. However, genetic testing, due to the large HCM genetic heterogeneity, is complex, time-consuming and expensive. Therefore, novel and sensitive diagnostic tests are needed for cascade family screening in order to identify HCM patients at an early stage.

Initial studies using tissue Doppler imaging (TDI) showed that mutation carriers without an overt HCM phenotype may have subtle myocardial diastolic dysfunction, as an early marker of the disease. [4]–[7] However, reported sensitivity and specificity of TDI to identify HCM mutation carriers were highly variable. [4], [5], [7] In addition, although an increased collagen synthesis has been demonstrated in HCM mutation carriers without a typical HCM phenotype, [8] the relation between early myocardial dysfunction and structural alterations remains unknown.

Ultrasonic tissue characterization with calibrated integrated backscatter (IBS) enables the evaluation of myocardial structural alterations in HCM patients, identifying the presence of myocardial disarray and diffuse myocardial fibrosis. [9] In addition, myocardial strain assessment based on two-dimensional (2D) speckle tracking analysis is a novel echocardiographic approach for a sensitive and angle-independent evaluation of myocardial global and regional systolic dysfunction. [10] The aim of this study was therefore to assess global and regional myocardial structural alterations (by calibrated IBS analysis) and systolic dysfunction (by speckle tracking strain analysis) in HCM mutation carriers without overt phenotype.

## Methods

### Patient Population and Protocol

A total of 16 unrelated HCM patients with an identified gene mutation referred to the cardio-genetic out-patient clinic of our Department were included and their 1^st^ degree relatives were offered genetic screening. The genetic testing protocol was approved by the Internal Review Board of our Institution (Leiden University Medical Center) and written informed consent was provided from all subjects undergoing genetic testing.

Pathogenic HCM gene mutations were found in 77 1^st^ degree relatives subsequently referred for detailed evaluation including clinical assessment, 12-lead electrocardiography (ECG), exercise testing, Holter monitoring and transthoracic echocardiography.

The echocardiographic examination included conventional LV measures, calibrated IBS for the assessment of myocardial structural alteration and 2D speckle tracking myocardial strain analysis. Ambulatory 24-hour ECG Holter monitoring was also performed in 67 (87%) 1^st^ degree relatives to document the presence of ventricular arrhythmias. Clinical and echocardiographic data were prospectively collected in electronic patient dossier (EPD-Vision version 8.3.3.6; Leiden, The Netherlands).

**Figure 1:**
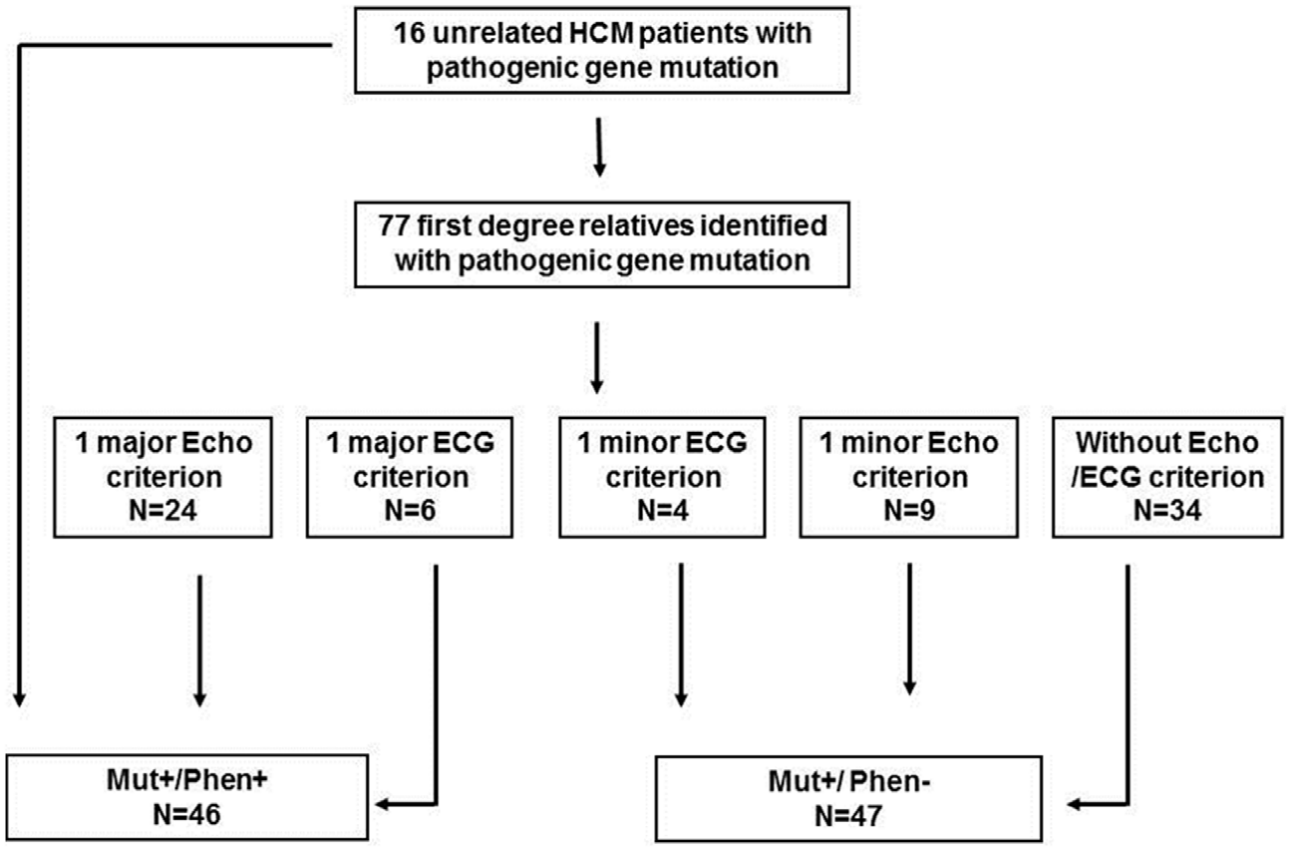
Schematic representation of the individual diagnosis of hypertrophic cardiomyopathy (HCM) within the study population, using the echocardiographic (Echo) and electrocardiographic (ECG) criteria proposed by McKenna et al^10^. The diagnosis of HCM is made in the presence of 1 major criterion or of 2 minor Echo criteria or of 1 minor Echo +2 minor ECG criteria. A total of 46 mutation carriers with phenotype expression (Mut+/Phen+) and 47 mutation carriers without phenotype expression (Mut+/Phen−) were identified.

The diagnosis of HCM was based on the criteria proposed by McKenna et al. for adult members of affected families, [2], [3], [11] which includes both echocardiographic and ECG criteria, for the identification of patients with emerging or mild HCM. As shown in Figure 1, a total of 30 relatives fulfilled the criteria for HCM phenotype and, together with the 16 index patients, were included in the phenotype positive group (Mut+/Phen+). The remaining 47 relatives were considered as mutation carriers without phenotype expression (Mut+/Phen−).

In addition, 25 individuals matched for age and gender with the Mut+/Phen− group were consecutively selected as a control group from an existing echocardiographic database with this information. These subjects were referred for atypical chest pain, palpitations, or syncope without murmur, and showed normal structural heart on echocardiography.

Subsequently, both myocardial structural alteration and systolic dysfunction were compared among these 3 groups.

### Standard Echocardiography

All patients were imaged using a commercially available system (Vivid 7, General Electric-Vingmed, Horton, Norway). Images were obtained using a 3.5-MHz transducer and digitally stored; offline analysis was performed using EchoPAC version 108.1.5 (General Electric – Vingmed). Maximum thickness of LV walls was measured in diastole from the basal, mid and apical short-axis views and from the parasternal long-axis view. [12] LV dimensions, LV volumes and ejection fraction were measured using the modified biplane Simpson’s rule. [13]

Evaluation of LV diastolic function was based on recommendation from the American Society of Echocardiography. [14] As previously described, [15] LV diastolic function was classified according to 4 categories: normal and diastolic dysfunction grade 1, 2, 3 and 4. Early diastolic velocity (È) of the lateral mitral valve annulus was measured by TDI and E/È ratio was calculated as a validated estimation of LV filling pressure. [16]

### Calibrated Integrated Backscatter (IBS)

**Figure 2:**
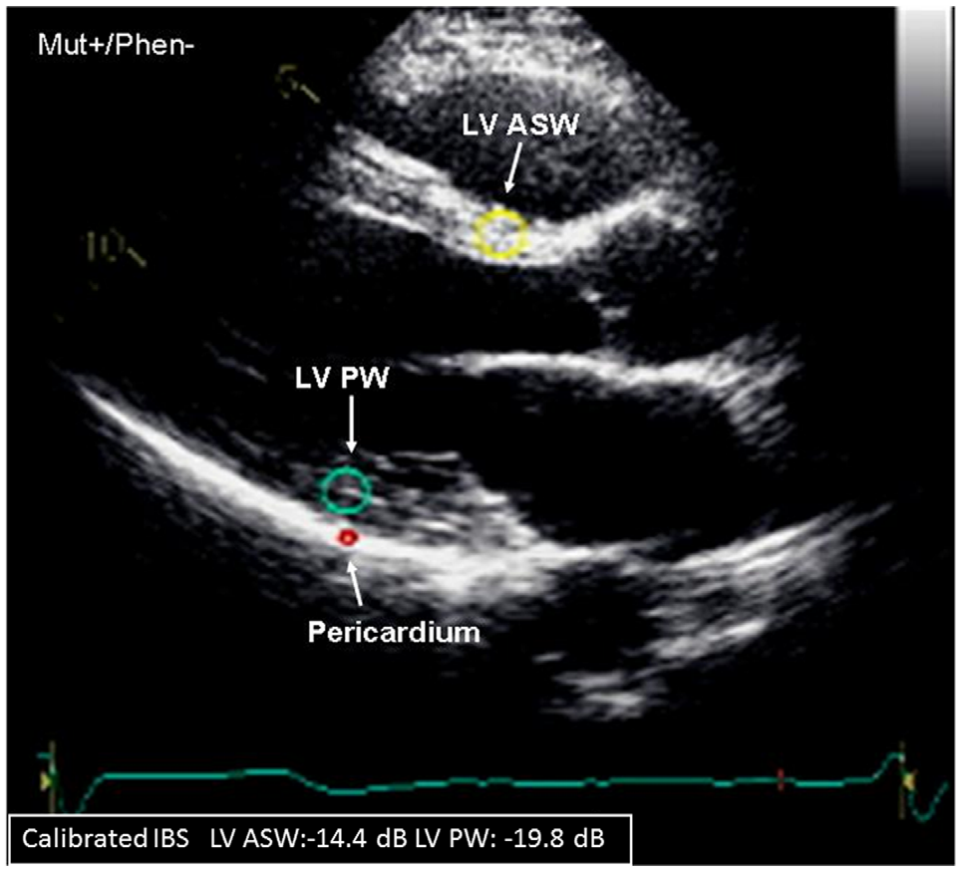
Assessment of left ventricular (LV) myocardial structural alteration by calibrated integrated backscatter (IBS) in a mutation carrier without HCM phenotype expression (Mut+/Phen−). A fixed region of interest is positioned at the level of the basal anteroseptal wall (ASW, yellow circle), of the basal posterior wall (PW, blue circle) and in the pericardium (red circle, for calibration). The basal ASW calibrated IBS of this Mut+/Phen− patient was −14.4 dB and the basal PW calibrated IBS was −19.8 dB, indicating an altered myocardial structure.

Calibrated IBS evaluates myocardial ultrasound reflectivity, providing an estimate of myocardial structural alterations and fibrotic content. [10], [17] The IBS measurements were obtained from the parasternal long-axis view. As shown in Figure 2, a 9×9 mm region of interest was positioned in the mid-myocardium of the basal segment of LV anteroseptal and posterior walls, and a 2×3-mm region of interest was positioned in the pericardium for reference. The measurements of IBS intensity were performed at end-diastole and expressed in decibels (dB). Calibrated IBS was calculated by subtracting pericardial IBS intensity from myocardial IBS intensity of the LV. A less negative value suggested a greater myocardial structural alteration.

### Two-dimensional Speckle Tracking Strain Analysis

**Figure 3:**
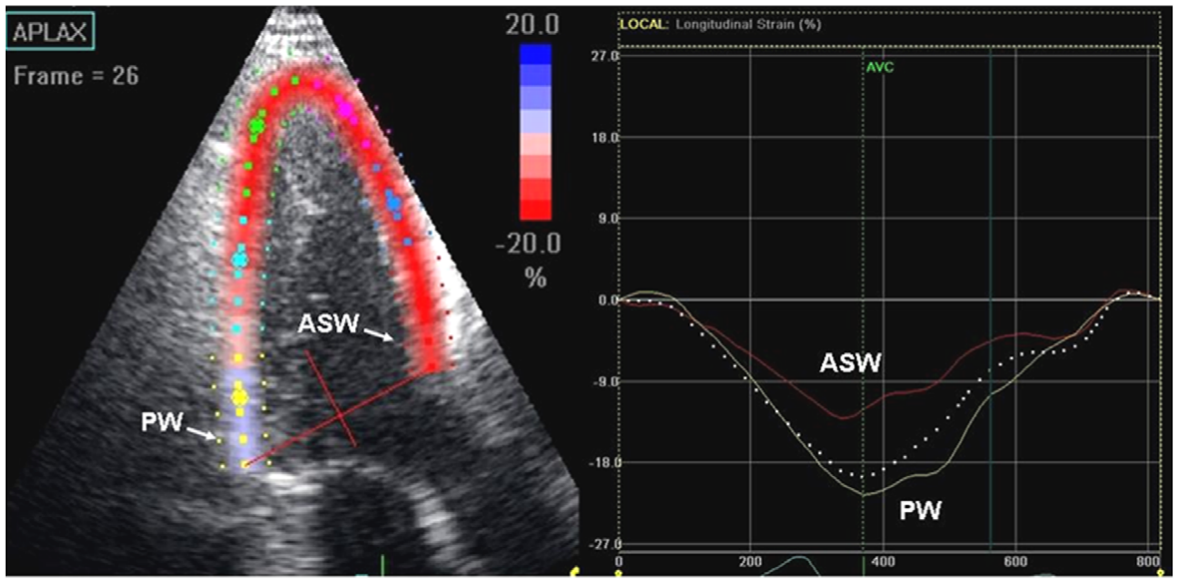
Example of impaired segmental strain measured by speckle tracking in the apical long-axis view (left panel), in a mutation carrier without HCM phenotype expression. The basal anteroseptal wall (ASW) is colored in red, while the basal posterior wall (PW) is colored in yellow. In the right panel, the corresponding longitudinal systolic strain curves: peak strain of basal ASW and PW are −14.8% and −24.8% respectively, suggesting segmental systolic dysfunction of the ASW with a preserved function of the PW. Conversely, global longitudinal strain (white dotted line) shows a normal value (−19.8%) in the apical long-axis view.

The measurement of global longitudinal, circumferential and radial strain using 2D speckle tracking analysis has been described previously. [18] Briefly, longitudinal strain was measured from 3 apical views (2 walls per view). Each wall was divided into 3 levels (basal, mid and apical) and subsequently 18 segmental strain curves were obtained. Global longitudinal strain was calculated as the average of peak systolic strain values of the 18 segments. In addition, peak longitudinal systolic strain of the basal segment of anteroseptal and posterior walls were evaluated as shown in Figure 3. These specific LV segments were chosen to allow a direct comparison with the IBS measurements (performed in the same regions) and to specifically explore the myocardial function of the basal anteroseptum, which is the predominant region of LV wall thickening. [19]

The global values of circumferential and radial strain were derived from the average of the peak systolic strain values obtained from the mid-ventricular short-axis view (divided into 6 segments).

### Statistical Analysis

Continuous variables are presented as mean(standard deviation). Categorical data are presented as frequencies and percentages. One-way analysis of variance (ANOVA) with post-hoc test by Bonferroni was used to examine the differences among groups. Receiver operator characteristic (ROC) curve analysis was performed to determine the accuracy of calibrated IBS, segmental longitudinal strain and È velocity in differentiating Mut+/Phen− patients from the controls. The optimal cut-off value was defined as the maximized value for the sum of sensitivity and specificity. To test the variability of calibrated IBS and regional longitudinal strain, 6 Mut+/phen− and 6 Mut+/Phen− patients were selected. A first observer analyzed a first dataset twice (within 1 month time) to determine intra-observer agreement. To test inter-observer variability, the measurements were repeated by a second experienced observer on the same dataset. The first observer also analyzed a second dataset acquired the same day of the first one in order to evaluate test-retest variability. Bland-Altman analysis was conducted (expressed as mean difference±2SD), and intraclass correlation coefficients (ICC) were calculated as indicators of reproducibility. All statistical analysis were performed using the statistical package SPSS for windows (Version 16.0, SPSS, Chicago, USA). A p value <0.05 was considered to be statistically significant.

## Results

### Clinical and Conventional Echocardiographic Characteristics

Clinical characteristics of all patients and normal controls are summarized in Table 1. The majority of Mut+/Phen− and Mut+/Phen+ patients had one of the Dutch founder mutations in the Myosin Binding Protein C (MYBPC3) gene (C2373dupG). [20] By design, age and gender were comparable between controls and Mut+/Phen− patients. In few Mut+/Phen− and Mut+/Phen+ patients, significant coronary artery disease was discovered during the clinical assessment; importantly, all patients were treated with complete revascularization and none of them had previous myocardial infarction or evidence of regional wall motion abnormalities.

**Table 1:** Baseline clinical characteristics of each group: controls, mutation carriers without phenotype expression (Mut+/Phen−), and mutation carriers with phenotype expression (Mut+/Phen+).

	Controls (n = 25)	Mut+/Phen− (n = 47)	Mut+/Phen+ (n = 46)
**Age, (SD) years**	38(16)	42(17)	52(15)
**Male, n (%)**	14 (56)	24(51)	32(78)*
**Identified mutation**			
**MYBPC3**			
C2373dupG, n (%)	−	33 (70)	31 (65)
C1483C>G, n (%)	−	2 (4)	3 (7)
C2827C>T, n (%)	−	2 (4)	3 (7)
C927-2A>G, n (%)	−	2 (4)	3 (7)
**MYH7**			
C1566T>A, n (%)	−	8 (17)	6 (13)
**Cardiovascular medication**			
ACEI, n (%)	1 (4)	0 (0)	11 (23)
ARB, n (%)	0 (0)	2 (4)	1 (2)
CCB, n (%)	0 (0)	2 (4)	15 (33)
Diuretics, n (%)	0 (0)	1 (2)	4 (9)
Beta-blockers, n (%)	0 (0)	3 (6)	19 (43)
**Cardiovascular risk factors**			
** **Hypertension	1 (4)	8 (17)	7 (15)
Diabetes mellitus	0 (0)	1(2)	1 (2)
Hyperlipidaemia	0 (0)	3 (6)	6 (13)
Smoking	4 (16)	3 (6)	2 (4)
Renal dysfunction	0 (0)	0 (0)	0 (0)
Coronary artery disease	0 (0)	3 (6)	1 (2)

Abbreviations: ACEI = angiotensin converting enzyme inhibitors; ARB = angiotensin receptor blocker; CCB = calcium channel blocker; MYBPC3 = myosin-binding protein C; MYH7 = β-myosin heavy chain.

* Significant difference vs. controls and Mut+/Phen−.

LV dimensions and ejection fraction were similar among all 3 groups (Table 2). The anteroseptal and posterior wall thickness in the Mut+/Phen+ group were the thickest among all 3 groups and were comparable between the Mut+/Phen− groups and controls. LV diastolic dysfunction was more common in the Mut+/Phen+ and Mut+/Phen− groups as compared with controls. In addition, È velocity was lower and LV filling pressures (estimated by E/È) were higher in the Mut+/Phen+ group than the other 2 groups. Importantly, È velocity was lower in the Mut+/Phen− group as compared to controls.

**Table 2:** Echocardiographic characteristics of each group: controls, mutation carriers without phenotype expression (Mut+/Phen−) and mutation carriers with phenotype expression (Mut+/Phen+).

	Controls(n = 25)	Mut+/Phen−(n = 47)	Mut+/Phen+(n = 46)	ANOVAp value
**LV Dimensions and systolic function**				
LVEDD (SD), cm	4.6(0.3)	4.4(0.6)	4.5(0.7)	0.09
LVESD (SD), cm	2.9(0.3)	2.9(0.4)	2.9(0.7)	0.81
Anteroseptal wall thickness (SD), cm	0.98(0.14)	1.15(0.15)	2.01(0.54)* †	**<0.01**
Posterior wall thickness (SD), cm	1.09(0.20)	1.14(0.22)	1.42(0.28)* †	**<0.01**
LVEF (SD), %	67(4)	65(7)	66(9)	0.16
LVEDV (SD), ml	77.3(18.0)	79.7(23.0)	88.2(25.2)	0.09
LVESV (SD), ml	24.0(6.1)	28.2(14.1)	30.7(10.3)	0.06
**Diastolic function**				
Grade:				**<0.01**
0, n (%)	20 (92.0)	35 (74.5)	20 (43.5)	
1, n (%)	2 (8.0)	12 (25.5)	19 (41.3)	
2, n (%)	0 (0)	0 (0)	4 (8.7)	
3–4, n (%)	0 (0)	0 (0)	3 (6.5)	
È velocity (SD), cm/s	10.7(2.3)	9.0(2.9)*	5.8(2.7)* †	**<0.01**
E/Èratio (SD)	7.4(1.6)	11.5(4.3)	17.3(7.8)* †	**<0.01**
**Speckle tracking**				
Global Longitudinal strain (SD), %	−21.3(1.3)	−20.9(3.3)	−15.2(3.7)* †	**<0.01**
Global Circumferential strain (SD), %	−21.2(3.7)	−21.2(3.7)	−15.9(3.6)* †	**<0.01**
Global Radial strain (SD), %	39.2(10.7)	37.4(18.6)	26.4(11.0)* †	**<0.01**

Abbreviations: LVEDD = left ventricular end-diastolic diameter; LVEDV = left ventricular end-diastolic volume; LVEF = left ventricular ejection fraction; LVESD = left ventricular end-systolic diameter; LVESV = left ventricular end-systolic volume; SD = standard deviation.

* Significant difference vs. Controls;

† Significant difference between Mut+/Phen− and Mut+/Phen+ groups.

### Calibrated IBS

**Figure 4:**
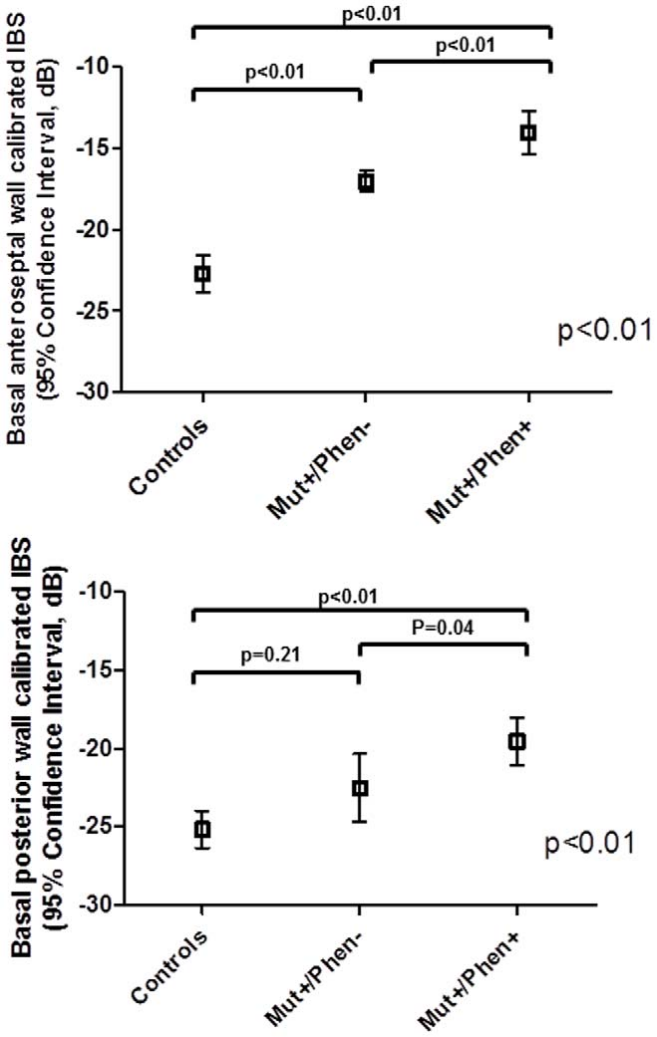
Calibrated integrated backscatter (IBS) of the basal anteroseptal and basal posterior walls in each group: controls, mutation carriers without phenotype expression (Mut+/Phen−) and mutation carriers with phenotype expression (Mut+/Phen+). Structural alterations of the basal anteroseptum are the greatest in the Mut+/Phen+ group, followed by Mut+/Phen− group and by controls. Similarly, structural alterations of the basal posterior wall are the greatest in the Mut+/Phen+ group, but are similar between the Mut+/Phen− group and controls.

As shown in Figure 4, structural alterations over the basal anteroseptal region evaluated by calibrated IBS were most predominant in the Mut+/Phen+ group (−14.0±4.6 dB) as compared to the Mut+/Phen− group (−17.0±2.3 dB; p<0.01) and controls (−22.6±2.9 dB; p<0.01). Importantly, the basal anteroseptal calibrated IBS of the Mut+/Phen− group was significantly higher as compared to controls (p<0.01). Also at the level of the basal posterior wall the degree of myocardial structural alterations assessed by calibrated IBS was the highest in the Mut+/Phen+ (−19.6±5.1 dB) group as compared with the Mut+/Phen− group (−22.5±7.4 dB, p = 0.04) and controls (−25.2±2.8 dB; p<0.01). However, the degree of myocardial structural alterations was not significantly different between Mut+/Phen− group and controls (p = 0.21).

### Speckle Tracking Strain Measurements

#### Global strain

Global longitudinal, circumferential and radial strain were significantly impaired in the Mut+/Phen+ group as compared to the other 2 groups. On the other hand, Mut+/Phen− group showed similar values as compared to controls (Table 2).

#### Segmental strain

**Figure 5:**
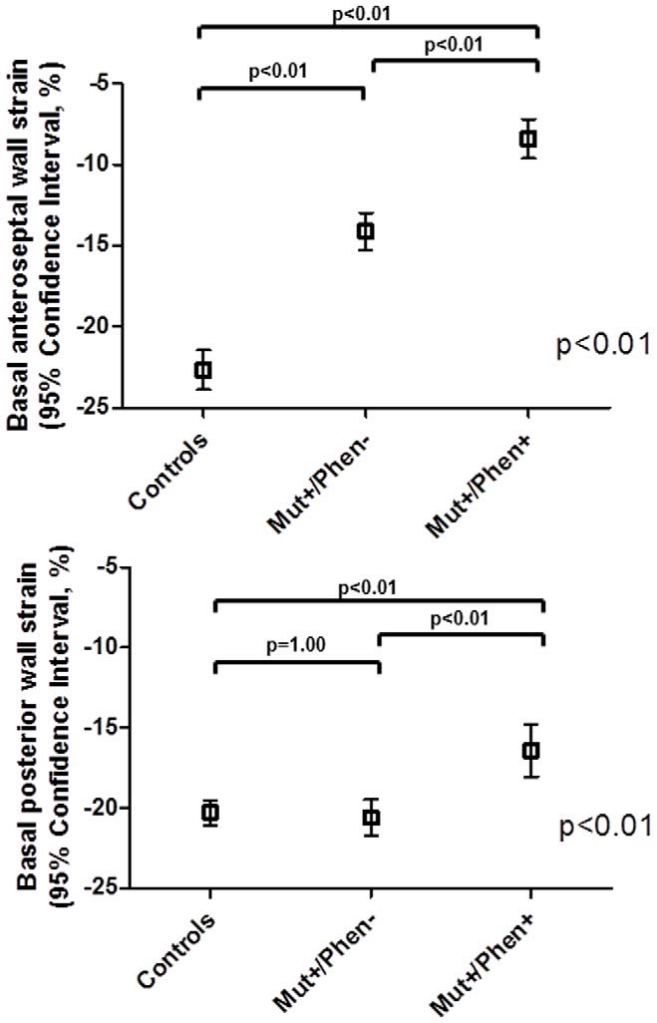
Segmental strain measured by speckle tracking of the basal anteroseptal and posterior walls in each group: controls, mutation carriers without phenotype expression (Mut+/Phen−) and mutation carrier with phenotype expression (Mut+/Phen+). Basal anteroseptal strain is the most impaired in Mut+/Phen+ group, followed by Mut+/Phen− group and controls. Basal posterior wall strain is the most impaired in Mut+/Phen+ group, while is similar between Mut+/Phen− group and controls.

As shown in Figure 5, regional strain analysis at the level of the basal anteroseptal and posterior walls was significantly different between the 3 groups. The basal anteroseptal longitudinal strain was significantly impaired in the Mut+/Phen+ group (−8.4±4.0%) as compared to both Mut+/Phen− group (−14.1±3.8%; p<0.01) and controls (−19.9±2.9%; p<0.01). Moreover, basal anteroseptal longitudinal strain of the Mut+/Phen− group was significantly lower as compared to controls (p<0.01).

Similarly, the basal posterior longitudinal strain was impaired in the Mut+/Phen+ group (−16.4±5.5%) as compared to the Mut+/Phen− group (−20.6±3.8%; p<0.01) and controls (−20.3±1.9%; p<0.01). However, no difference was noted in the basal posterior longitudinal strain between Mut+/Phen− group and controls.

### Calibrated IBS and Segmental Speckle Tracking Strain Measurements to Predict Mutation Carriers

**Figure 6:**
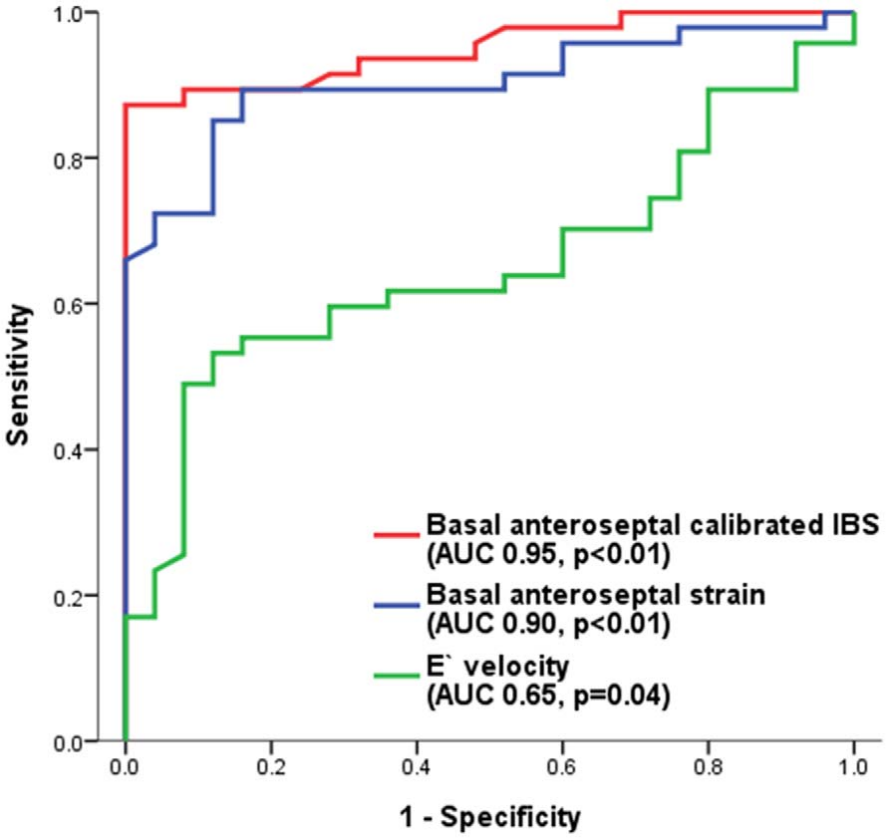
Receiver operator characteristic curve analysis to determine the accuracy of basal anteroseptal calibrated integrated backscatter (IBS), and longitudinal strain and È velocity to differentiate Mut+/Phen− patients from controls. Basal anteroseptal calibrated IBS >−19.0 dB yielded a sensitivity of 89% and a specificity of 84% to differentiate Mut+/Phen− patients from controls. Basal anteroseptal strain >−18.0% yielded a sensitivity of 85% and specificity of 84%. È velocity <10 cm/s provided a modest sensitivity of 62% and specificity of 60% to differentiate Mut+/Phen− patients from controls. AUC = area under the curve.

The ROC curve was generated to determine the accuracy of basal anteroseptal calibrated IBS, basal anteroseptal strain and È velocity measurements to differentiate the Mut+/Phen− group from controls (Figure 6). All 3 parameters significantly differentiated Mut+/Phen− group from controls, but the area under the curve was higher for the basal anteroseptal calibrated IBS and strain as compared to È velocity. A cutoff value of >−19.0 dB for basal anteroseptal calibrated IBS provided a sensitivity of 89% and specificity of 84%, while a cutoff value of >−18.0% for basal anteroseptal strain showed a sensitivity of 85% and specificity of 84%. For È velocity, a cut-off value of <10 cm/s yield a modest sensitivity and specificity (62% and 60%, respectively).

A combination of >−19.0 dB basal anteroseptal calibrated IBS or >−18.0% basal anteroseptal strain had a sensitivity of 98%, a specificity of 72%, a positive predictive value of 87% and a negative predictive value of 95%.

### Holter Results and Clinical Outcomes in Mutation Carriers

Holter ECG recordings were analyzed taking into account the percentage of premature ventricular contractions and the presence of non-sustained ventricular tachycardia (NSVT) defined as 3 or more consecutive ventricular beats at a rate of >120 beats/min, lasting for <30 s. Among the 47 Mut+/Phen− patients, 43(91%) underwent ECG Holter assessment and 9 patients showed abnormal results defined by premature ventricular contractions >100 per 24 hours (6 patients) and NSVT (3 patients). [21]

A greater extent of myocardial structural alterations in the basal anteroseptal wall, estimated by calibrated IBS (−15.3±3.1 dB), was observed in patients with abnormal Holter results as compared to those with normal results (17.4±1.7 dB, p<0.01). However, no significant difference was observed for calibrated IBS of the basal posterior wall between these 2 groups (−22.4±2.6 vs.−22.5±8.1 dB, p = 0.96). Moreover, the segmental strain over the basal anteroseptal wall (−13.2±2.6 vs.−14.23±4.0%, p = 0.47) and basal posterior wall (−21.0±4.5 vs.−20.5±3.7%, p = 0.69) were also similar between these 2 groups.

### Reproducibility

Inter-observer and intra-observer variability were evaluated by two independent observers. The mean differences for intra-observer variability for basal anteroseptal calibrated IBS was 0.1±0.2 dB (p = 0.72, ICC = 0.99), for basal posterior calibrated IBS was 0.2±0.4 dB (p = 0.78, ICC = 0.99), for basal anteroseptal strain was 0.2±0.4% (p = 0.17, ICC = 0.99) and for basal posterior anteroseptal strain was 0.2±1.1% (p = 0.19, ICC = 0.99).

Mean absolute differences for inter-observer variability for basal anteroseptal calibrated IBS was 0.5±1.5 dB (p = 0.09, ICC = 0.99), for basal posterior calibrated IBS was 0.3±1.2 dB (p = 0.35, ICC = 0.98), for basal anteroseptal strain was 0.6±2.0% (p<0.01, ICC = 0.97) and for basal posterior strain was 1.1±1.0% (p = 0.35, ICC = 0.99).

Test-retest reproducibility of calibrated IBS and segmental strain were evaluated using a second echocardiographic dataset acquired the same day. Mean absolute differences and coefficients of variations between the 2 examinations were as follows: basal anteroseptal calibrated IBS 0.4±0.2 dB p<0.01, ICC = 0.99); basal posterior calibrated IBS 0.3±0.7 dB (p<0.01, ICC = 0.99); basal anteroseptal strain 0.5±0.9% (p<0.01, ICC = 0.99); basal posterior strain 0.7±0.9% (p<0.01, r = 0.99).

## Discussion

The results of the current study demonstrated that calibrated IBS and 2D speckle tracking derived strain are able to detect myocardial regional structural and functional abnormalities in HCM mutation carriers without phenotype expression. In particular, the combined measure of basal anteroseptal calibrated IBS and longitudinal strain provided a highly sensitive index to distinguish mutation carriers from normal controls.

### Global LV Dysfunction in Mut+/Phen− Patients

The advent of genetic testing has allowed the detection of pathogenic gene mutations in family members of HCM patients before the development of LVH or any HCM phenotype. In a high percentage of HCM patients, the development of LVH is delayed late into adulthood, especially in families with myosin-binding protein C mutation. [2] However, genetic testing might not be widely available due to the complex, time-consuming and expensive laboratory techniques required. In addition, not all (young) patients are motivated to have genetic analysis performed, as financial institutions may limit their eligibility to secure loans and insurance policies, even though they do not have a HCM phenotype at that moment.

Therefore, several studies, using either echocardiography or magnetic resonance imaging [4]–[7], [22], have focused on the ability to detect subtle myocardial dysfunction, which could be applied to identify HCM mutation carriers without overt LVH. These reports have demonstrated that mutation carriers without LVH present an impaired LV diastolic function (mainly measured by TDI) as compared with normal controls. [4]–[7] Similarly, the current study showed that Mut+/Phen− patients had more frequent LV diastolic dysfunction and significantly lower È velocity as compared to controls. However, conflicting results are reported regarding the sensitivity and specificity of this approach (due to a significant overlap with normal values) and a definition of an accurate cut-off value to distinguish HCM mutation carriers versus normal controls is lacking. [4], [5], [7] Similarly in the current study, È velocity showed only modest sensitivity and specificity to differentiate Mut+/Phen− patients from controls.

Besides the assessment of LV diastolic function, Ho and colleagues have also evaluated LV global systolic function using novel strain and strain rate imaging. [6] In their study, preclinical HCM mutation carriers showed preserved LV global strain and strain rate as compared to controls, while overt HCM patients showed impaired LV global strain and strain rate, despite normal LVEF and dimensions. Similarly in the present study, Mut+/Phen− patients showed values of LV global longitudinal, radial and circumferential strain comparable to normal subjects. Therefore, assessment of LV global systolic function seems not able to distinguish between HCM mutation carriers and normal controls.

### Segmental Myocardial Structural Alterations and Dysfunction in Mut+/Phen− Patients

The present study showed that Mut+/Phen− patients had a significant increased calibrated IBS of the basal anteroseptal region as compared to controls, suggesting substantial myocardial structural alterations. The altered IBS values of the basal anteroseptal region might reflect an increased myocardial interstitial fibrosis in these patients, as recently suggested by Ho et al., who demonstrated increased myocardial type I collagen synthesis in HCM mutation carriers without overt LVH. [9] However, the authors did not observe any significant area of myocardial fibrosis as assessed by delayed-enhancement magnetic resonance imaging (MRI). An explanation for these findings might be the limited resolution of this imaging technique to identify fine interstitial fibrosis, especially over the basal anteroseptal region. [19]

In addition, ultrasonic techniques are sensitive to anisotropic myocardial architecture, such as myocardial disarray (myocytes arranged at oblique and perpendicular angles to each other), which characterizes the myocardium of HCM patients. [23], [24] Mizuno and colleagues showed that calibrated IBS significantly correlated with the degree of myocardial disarray and interstitial fibrosis assessed by endomyocardial biopsy in HCM patients. [9] Although the specificity may be lower than delayed-enhancement MRI, calibrated IBS might provide a more sensitive technique to assess subtle myocardial structural alterations in HCM mutation carriers.

In addition to the structural abnormalities detected by calibrated IBS, the current study showed that Mut+/Phen− patients have also impaired segmental longitudinal strain in the basal anteroseptal region, but not in the basal posterior region. The preserved LV global strain values observed in the present study in Mut+/Phen− patients might be the result of compensation from the remaining myocardial segments with normal function.

The subtle segmental structural and functional alterations circumscribed to the anteroseptum observed in Mut+/Phen− patients can be explained by the preferential involvement of the basal anteroseptal region by LVH in HCM disease. Previous studies showed that in up to 96% of HCM patients, the anterior portion of the ventricular septum is the most common site with LVH as assessed by echocardiography. [25] In addition, a report by Germans et al. has demonstrated using MRI that 81% of mutation carriers had crypts located in the basal and mid septum up to the subepicardium region. [26] Similarly, a recent report using also MRI showed that hypertrophy usually involves the LV with a segmental rather than global pattern, with the basal anterior free wall and ventricular septum being the most frequently affected. [19] Segmental subtle functional and structural alterations in mutation carriers may therefore anticipate the development of hypertrophy in the same LV region, while global LV function remains normal. In vitro studies have in fact demonstrated that sarcomere protein mutations are associated with impaired myocardial contractility, supporting the hypothesis that subtle myocardial dysfunction precedes and promotes the development of hypertrophy. [4], [27], [28] However, further longitudinal studies are needed to describe the sequential phenotypic changes of HCM patients.

Importantly, the current study showed that combined measurements of basal anteroseptal calibrated IBS or longitudinal strain have a high sensitivity and negative predictive value to distinguish Mut+/Phen− patients versus normal controls, suggesting a new tool helping to identify subjects without mutation among familial of HCM patients.

### Clinical Outcome of Mut+/Phen− Patients

Recent reports have suggested that mutation carriers without clinical overt HCM frequently have risk factors for sudden cardiac death. [29], [30] In particular, NSVT occurred in 2.2–5.6% of mutation carriers without clinical HCM. [29]–[31] Similarly in the present cohort, NSVT detected by ECG Holter monitoring was noted in 3 Mut+/Phen− patients. These observations suggested that mutation carriers are also at risk of developing ventricular arrhythmias, even before the development of significant LVH. A recent study by Christiaans et al. nonetheless showed that mutation carriers have a low risk of sudden cardiac death before the presence of LVH. [31] Therefore, future studies are required to further explore the clinical characteristics as well as prognostic outcome of these patients.

### Limitations

Majority of patients in the current study had a mutation in the MYBPC3 gene and therefore results cannot be extrapolated to other groups of mutations (e.g. TNNT2, TPM1). Furthermore, although the most likely mechanism behind an increased cIBS in this population is the presence of myocardial structural alterations, histopathological examination of the myocardium was not available in the studied patients and the exact mechanism generating the cIBS signals should be clarified in further specific studies. In addition, the potential variation of cIBS and segmental strain affected by age and coexisting disease, such as hypertension and underlying coronary artery disease, could not be fully evaluated due to the small study population and the relatively low prevalence of these potential confounders. Larger study populations involving HCM familial screening would be required to further delineate the sensitivity and specificity of these techniques to predict the presence of mutation and to confirm the intra- and inter-observer reproducibility on a multi-center setting. In particular, a specific evaluation should be performed to better assess test-retest reproducibility, including multiple examinations over time, and to explore possible differences among vendors. Lastly, prospective follow-up is necessary to evaluate the clinical significance of altered basal anteroseptal calibrated IBS and longitudinal strain in Mut+/Phen− patients.

### Conclusion

Novel echocardiographic assessments by calibrated IBS and speckle tracking derived strain allow detection of subtle myocardial structural alterations and impaired systolic function in mutation carriers without HCM phenotype. Importantly, the preferential involvement of the basal anteroseptum demonstrated a segmental rather than global involvement of the myocardium in this group of patients. These findings allow identification and monitoring of patients with HCM gene mutations before the development of LVH.
